# L(+) and D(−) Lactate Are Increased in Plasma and Urine Samples of Type 2 Diabetes as Measured by a Simultaneous Quantification of L(+) and D(−) Lactate by Reversed-Phase Liquid Chromatography Tandem Mass Spectrometry

**DOI:** 10.1155/2012/234812

**Published:** 2012-03-08

**Authors:** Jean L. J. M. Scheijen, Nordin M. J. Hanssen, Marjo P. H. van de Waarenburg, Daisy M. A. E. Jonkers, Coen D. A. Stehouwer, Casper G. Schalkwijk

**Affiliations:** ^1^Laboratory for Metabolism and Vascular Medicine, Department of Internal Medicine, Maastricht University, P. Debeyelaan 25, P.O. Box 5800, 6202 AZ Maastricht, The Netherlands; ^2^Cardiovascular Research Institute Maastricht, Maastricht University, Maastricht, The Netherlands; ^3^Division of Gastroenterology and Hepatology, Department of Internal Medicine, Maastricht University, Maastricht, The Netherlands

## Abstract

*Background*. Plasma and urinary levels of D-lactate have been linked to the presence of diabetes. Previously developed techniques have shown several limitations to further evaluate D-lactate as a biomarker for this condition. *Methods*. D- and L-lactate were quantified using ultraperformance liquid chromatography tandem mass spectrometry with labelled internal standard. Samples were derivatized with diacetyl-L-tartaric anhydride and separated on a C_18_-reversed phase column. D- and L-lactate were analysed in plasma and urine of controls, patients with inflammatory bowel disease (IBD), and patients with type 2 diabetes (T2DM). *Results*. Quantitative analysis of D- and L-lactate was achieved successfully. Calibration curves were linear (*r* ^2^ > 0.99) over the physiological and pathophysiological ranges. Recoveries for urine and plasma were between 96% and 113%. Inter- and intra-assay variations were between 2% and 9%. The limits of detection of D-lactate and L-lactate in plasma were 0.7 *μ*mol/L and 0.2 *μ*mol/L, respectively. The limits of detection of D-lactate and L-lactate in urine were 8.1 nmol/mmol creatinine and 4.4 nmol/mmol creatinine, respectively. Plasma and urinary levels of D- and L-lactate were increased in patients with IBD and T2DM as compared with controls. *Conclusion*. The presented method proved to be suitable for the quantification of D- and L-lactate and opens the possibility to explore the use of D-lactate as a biomarker.

## 1. Introduction

There are several conditions in which D-lactate can become increased in blood and urine in humans [1]. Recent studies demonstrated increased levels of D-lactate in diabetes and in infection, ischemia, and trauma, suggesting the use of D-lactate as a biomarker. However, to further explore the use of D-lactate as such a biomarker, there is a need of an improved method for analysing D-lactate.

**Figure 1:**
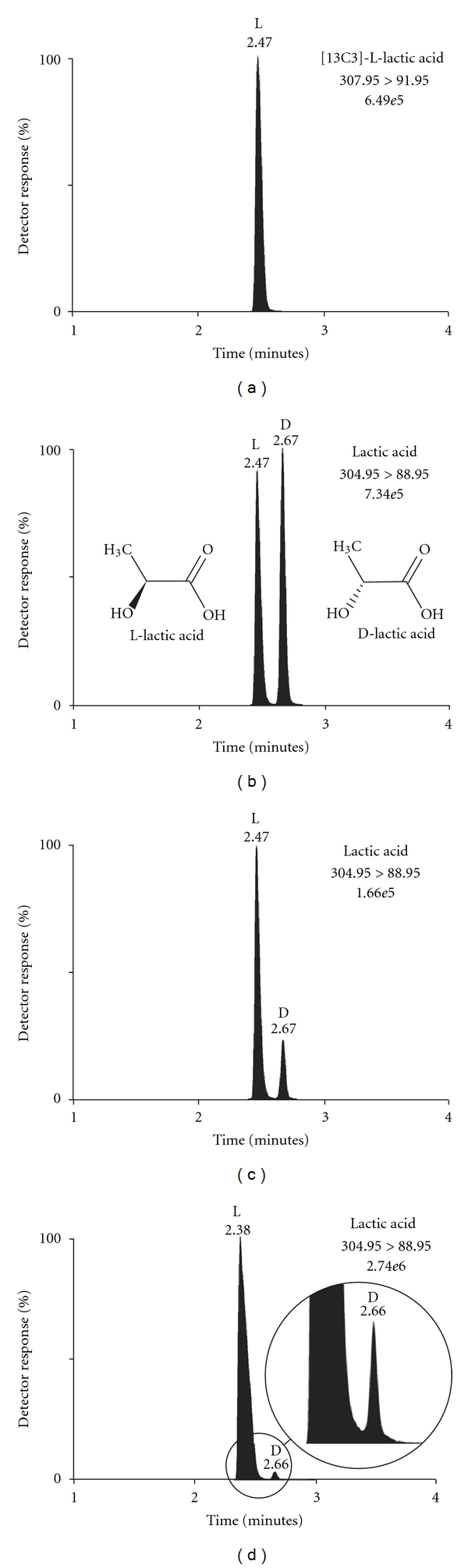
Representative chromatograms of D- and L-lactic acid derivatives^(a)^. (a) Internal standard [^13^C_3_]-L-lactate chromatogram (435 *μ*mol/L, 72 pmol). (b) Representative chromatogram of a standard solution of D- and L-Lactate (351 *μ*mol/L, 58.2 pmol, 501 *μ*mol/L, and 83.0 pmol, resp.) and molecular structures of optical isomers L- and D-lactates. (c) Representative chromatogram of a urine sample (D- and L-Lactate; 14.1 *μ*mol/L, 2.3 pmol, 74.6 *μ*mol/L, and 12.4 pmol, resp.). (d) Representative chromatogram of a plasma sample (D- and L-Lactate; 11.2 *μ*mol/L, 1.9 pmol, 1375.0 *μ*mol/L, and 227.7 pmol, resp.). ^(a)^Injection volume: 2 *μ*L.

Lactate has two optical isomers, L-lactate and D-lactate (Figure 1(b)). L-lactate is the most abundant enantiomer of lactate. It is formed mainly during anaerobic glycolysis by conversion of pyruvate to L-lactate by lactate dehydrogenase [2]. D-lactate is often considered as the nonphysiological counterpart of L-lactate [1]. Under physiologic conditions, the concentration of D-lactate is a 100-fold lower when compared to L-lactate [3]. The origin of D-lactate in human metabolism is thought to be derived from two major sources, namely, degradation of methylglyoxal into D-lactate by the glyoxalase pathway and production by intestinal bacteria. Indeed, disturbances in these metabolic pathways are associated with increased levels of D-lactate [3–7]. Although some enzymes capable of metabolizing D-lactate have been described [8], its metabolism is very inefficient, and D-lactate is mainly excreted in urine [1].

Methylglyoxal is a highly reactive compound formed in the process of glycolysis and lipid peroxidation. Methylglyoxal is increased in diabetes and is a major precursor in the formation of advanced glycation endproducts [9]. Methylglyoxal is degraded by the glyoxalase system resulting in D-Lactate. D-lactate in plasma and urine has been shown to be increased in patients with diabetes [3, 7]. D-lactate can be used as a reflection of methylglyoxal and is much easier to measure than the very reactive methylglyoxal.

In the colon, many commensal bacteria produce D-lactate as a result of anaerobic glycolysis. Under physiological circumstances, this D-lactate is further metabolized by the commensal bacteria to acetate. Therefore, D-lactate produced in the intestinal tract does not significantly contribute to levels of D-lactate in the systemic circulation under physiological circumstances [10]. However, under pathologic conditions, systemic D-lactate levels may rise due to intestinal production by bacteria. In patients with ulcerative colitis, gut ischemia, and appendicitis, increased levels of D-lactate have indeed been demonstrated [4–6]. The most extreme example of impaired gut permeability and bacterial overgrowth is short bowel syndrome, which is associated with D-lactate acidosis [10].

So far, D- and L-Lactate have been analysed by several different techniques ranging between chiral stationary phase liquid chromatography using UV or fluorescence detection [3, 11–15], enzymatic assays [7, 16–21], gas chromatography mass spectrometry (GC/MS) methods [22, 23], liquid chromatography mass spectrometry (LC/MS) methods [24, 25], and reversed phase liquid chromatography using fluorescence detection [26]. However, these techniques have several shortcomings such as low sensitivity [11, 24, 27], large sample volume [19, 21, 22], complex chromatographic systems [3, 12, 13], and long run times [3, 11, 26, 27]. To further explore the use of D-lactate as a biomarker, there is a need of an improved method for analysing D-lactate.

In this paper, we describe a highly sensitive, specific and fast ultraperformance liquid chromatography (UPLC) tandem mass spectrometry (MS/MS) method for the analysis of D- and L-Lactate in plasma and urine without the need of a chiral stationary phase. We achieved a significant improvement over the methods described in the literature and obtained a strong tool for the analysis of D- and L-Lactate in large studies. With this method, we measured plasma and urine concentrations of D- and L-Lactate in controls, patients with inflammatory bowel disease (IBD), and in patients with type 2 diabetes (T2DM).

## 2. Materials and Methods

### 2.1. Chemicals and Reagents

L(+)-lactate (98%) and dichloromethane (≥99.9%) were obtained from Sigma-Aldrich. Ammonia solution (25%) and acetic acid anhydrous (100%) were obtained from Merck. Formic acid (p.a.), (+)-O,O′-diacetyl-L-tartaric anhydride (≥97%) (DATAN) and lithium D-lactate (≥99%) were obtained from Fluka. Water and acetonitrile (ULC-MS grade) were obtained from Biosolve. [^13^C_3_]-Sodium L-lactate (20%, w/w in water) was obtained from Cambridge Isotope Laboratories.

### 2.2. Chromatographic Conditions

Samples were analysed by reversed phase LC-tandem MS using an Acquity UPLC BEH C_18_ analytical column (100 × 2.1 mm, 1.7 *μ*m, Waters). Detection was carried out using a Xevo TQ tandem mass spectrometer (Waters), which was operated in negative multiple-reaction-monitoring (MRM) mode. UPLC analysis was performed using a binary gradient at a flow of 0.5 mL/min using an Acquity UPLC (Waters). Solvent A was 1.5 mM ammonium formate (pH = 3.6), and solvent B was acetonitrile. A linear gradient was started at 99.5% solvent A, which was changed within 3 minutes to 97% solvent A. After cleaning the column with 40% solvent B during 2 minutes, the column was equilibrated for 1 minute at the initial composition. Injection volume was 2 *μ*L, and column temperature was set at 31°C. Samples were kept at 6°C. Chromatograms were acquired and processed with Masslynx V4.1 SCN 644 (Waters).

### 2.3. Mass Spectrometry Conditions

MRM transitions were optimised using direct infusion of D-lactate (500 *μ*mol/L), [^13^C_3_]-L-Lactate (400 *μ*mol/L), and L-lactate (1000 *μ*mol/L) standard solution into the tandem MS at a flow of 20 *μ*L/min. Optimal conditions for all parents were found at a capillary voltage of 1.5 kV and a cone voltage of 10 V. The source and desolvation temperature were 150 and 450°C, respectively. The cone gas flow and desolvation gas flow were 0 and 800 l/hour, respectively. To establish the most sensitive daughter ions, the collision energy was set at 8 eV with a collision gas flow of 0.15 mL/min.  Table 1 shows the optimised MRM settings.

### 2.4. Plasma and Urine Samples

Three groups were selected for D- and L-Lactate measurements. Diabetic individuals and nondiabetic controls were sex- and age-matched subsets recruited from the cohort study of diabetes and atherosclerosis in Maastricht (CODAM). The characteristics of these subjects have been described in detail elsewhere [28]. In short, the control group (*n* = 52 ) was 55.8 ± 0.7 years old, 46% female, and had an HbA1C of 5.6 ± 0.1 % and a fasting plasma glucose of 5.2 ± 0.1  mmol/L. Group 2, the patients with T2DM (*n* = 52 ), were 56.3 ± 0.6 years old, 39% female, and had an HbA1C of 6.9 ± 0.2 % and fasting plasma glucose of 8.0 ± 0.2  mmol/L. Group 3 consisted of patients with IBD in remission: 32 plasma samples (52.3 ± 8.6 years, 44% female) and 34 urine samples (54.6 ± 14.1 years, 59% female). These samples were recruited from the outpatient-clinic of the Maastricht University Medical Center.

For comparison of the proposed UPLC tandem MS method with the enzymatic method, we analysed plasma and urinary D-lactate, with both methods, in rat samples. These animals were described in details elsewhere [29].

### 2.5. Plasma Sample Preparation

To 25 *μ*L of internal standard solution (containing 434.75 *μ*mol/L [^13^C_3_]-L-lactate) 25 *μ*L of plasma was added. Samples were mixed thoroughly and subsequently deproteinized with 600 *μ*L of a mixture of methanol : acetonitrile (1 : 1, by volume) and centrifuged at room temperature during 10 minutes at 14000 rpm. The supernatant was pipetted into a reaction vial and evaporated to dryness under a gentle stream of nitrogen at a temperature of 50°C. Fifty microliters of freshly made DATAN (50 mg/mL dichloromethane : acetic acid (4 : 1, by volume)) was added. The vial was capped, vortexed, and heated at 75°C for 30 minutes. After 30 minutes, the vial was allowed to cool down to room temperature, and the mixture was evaporated to dryness with a gentle stream of nitrogen. The derivatized residue was reconstituted with 150 *μ*L acetonitrile : water (1 : 2, by volume).

### 2.6. Urine Sample Preparation

Twenty five microliters of internal standard solution (containing 434.75 *μ*mol/L [^13^C_3_]-L-lactate), 25 *μ*L urine, and 300 *μ*L of methanol were pipetted into a reaction vial. Samples were mixed thoroughly and evaporated to dryness under a gentle stream of nitrogen at a temperature of 50°C. Fifty microliters of freshly made DATAN (50 mg/mL dichloromethane : acetic acid (4 : 1, by volume)) was added. The vial was capped, vortexed, and heated at 75°C for 30 minutes. After 30 minutes, the vial was allowed to cool down to room temperature, and the mixture was evaporated to dryness with a gentle stream of nitrogen. The derivatized residue was reconstituted with 300 *μ*L acetonitrile : water (1 : 2, by volume).

### 2.7. Method Validation

Linearity of the detection of D- and L-Lactate was tested in water and matrix by adding D- and L-Lactate standard to water and during preparation of plasma or urine samples (Table 2). Calibration curves were obtained by linear regression of a plot of the analyte concentration (*x*) versus the peak-area ratio of the analyte/internal standard area (*y*). For both the analytes, [^13^C_3_]-L-lactate was used as internal standard.

The lower limit of detection was determined by calculating the concentration at a signal-to-noise ratio of six (s/N: 6, injection volume: 2 *μ*L).

For recovery experiments, standard solutions of D- and L-Lactate were added to urine or plasma and subsequently prepared as described in the sample preparation section.

The intra-assay variation of the method was determined in two different plasma and urine samples (*n* = 10) analysed in one batch during one day. The interassay variation of the method was determined in two different plasma and urine samples divided into batches and analysed during 10 different days.

Freeze-thaw stability was tested in two different plasma and urine samples by snap-freezing these samples in liquid nitrogen and thawing them for 5 subsequent cycles.

To investigate the stability of plasma and urine samples, stored at 6°C in the autosampler, replicate injections of two different plasma and urine samples were done every hour during 24 hours.

### 2.8. Determination of Fasting Plasma Glucose, Hba1C, and Urinary Creatinine

After an overnight fast, plasma glucose concentrations (mmol/L) were measured with a hexokinase glucose-6 phosphate dehydrogenase method (ABX Diagnostics, Montpellier, France). Hba1C (%) was determined by ion-exchange high-performance liquid chromatography (HPLC) (Bio-Rad, Veenendaal, the Netherlands). Fasting plasma glucose concentrations and Hba1C were determined in the CODAM participants only.

Urinary D- and L-Lactate concentrations were expressed as *μ*mol/mmol creatinine. Creatinine concentration in urine was analysed using a Beckman LX20 analyser (Beckman Coulter) based on the Jaffé reaction method [30].

### 2.9. D-Lactate Enzymatic Assay

For method comparison, an enzymatic-spectrophotometric method, based on the oxidation of D-lactate to pyruvate by NAD^+^ in the presence of bacterial D-lactate hydrogenase, was used [21].

### 2.10. Statistical Analysis

The method validation data were expressed as mean and standard deviation (SD). To investigate agreement between the enzymatic and UPLC tandem MS method, we used linear regression and a Bland-Altman plot after log normalisation of the rat urine samples. Limits of agreement were defined as 2 times the SD. The patient study data were expressed as mean and standard error of the mean (SEM). To detect group differences, we applied analysis of variance (ANOVA) with post hoc Bonferroni correction. *P* value <0.05 was considered statistically significant.

## 3. Results

### 3.1. Reversed Phase Chromatography

D- and L-Lactate DATAN derivatives yielded a baseline separation on a reversed phase UPLC column with a retention time of 2.7 minutes for D-lactate and 2.5 minutes for L-lactate. Representative chromatograms are shown in Figure 1.

### 3.2. Stability of D- and L-Lactate

After 5 freeze-thaw cycles no change of D- and L-Lactate levels was observed, as tested in two different plasma and urine samples (data not shown).

To make large number of measurements within one run possible, we tested the stability of D- and L-Lactate when samples were stored in the autoinjector at 6°C. D- and L-Lactate were at least stable for 24 hours, and no degradation was observed after replicate injections of two different plasma and urine samples (data not shown).

### 3.3. Linearity, Lower Limit of Detection, Recovery, and Precision

Linearity of the detection of D- and L-Lactate was tested in matrix and water. For plasma, the slope, tested in three different plasma samples and in water measured on different days, was 1.4 ± 6.3 % (mean ± CV%) for D-lactate and 0.75 ± 3.2 % for L-lactate (Table 2). For urine, the slope, tested in three different urine samples and in water measured on different days, was 1.09 ± 4.0 % for D-lactate and 0.80 ± 1.6 % for L-lactate (Table 2). The limits of detection of D-lactate and L-lactate in plasma were 0.65 *μ*mol/L (108 fmol) and 0.2 *μ*mol/L (33 fmol), respectively. The limits of detection of D-lactate and L-lactate in urine were 8.1 nmol/mmol creatinine (40 fmol) and 4.4 nmol/mmol creatinine (22 fmol); respectively.

We found recoveries, for urine and plasma, between 96% and 113% (Table 3). The validation data demonstrated inter- and intra-assay variations between 2% and 9% (Table 4).

### 3.4. Comparison of the UPLC Tandem MS Method versus the Enzymatic Assay

**Figure 2:**
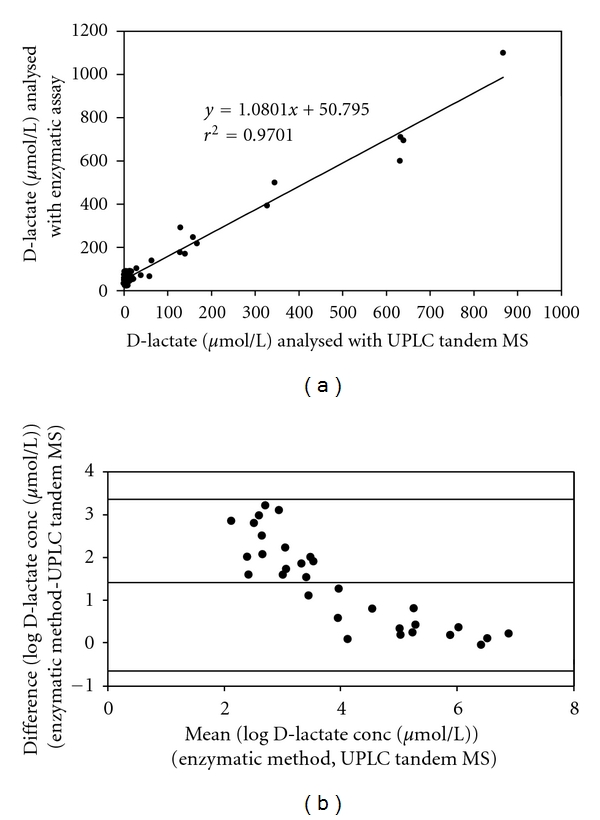
Comparison of urinary D-lactate in rat urine as measured by UPLC tandem MS and enzymatic method. (a) Correlation between D-lactate levels measured by UPLC tandem MS and enzymatic method. (b) Bland-Altman plot of log-transformed D-lactate levels as measured by UPLC tandem MS and enzymatic method.

We compared the proposed UPLC-tandem MS method with the enzymatic assay, by analysing plasma and D-lactate levels in rat urine with both methods. However, due to low D-lactate levels in plasma, it was not possible to analyse these samples with the enzymatic assay (data not shown). For urine, linear regression of the data resulted in the equation *y* = 1.08*x* + 50.795, with excellent correlation (*r* = 0.985) between both techniques (Figure 2(a)). However, the Bland Altman plot showed that although in the higher range both techniques are in excellent agreement, in the lower range considerately higher values were measured with the enzymatic technique as compared with the UPLC tandem MS method (Figure 2(b)).

### 3.5. Comparison of Urinary and Plasma D- and L-Lactate Concentration between Controls, Individuals with T2DM, and Individuals with IBD

**Figure 3:**
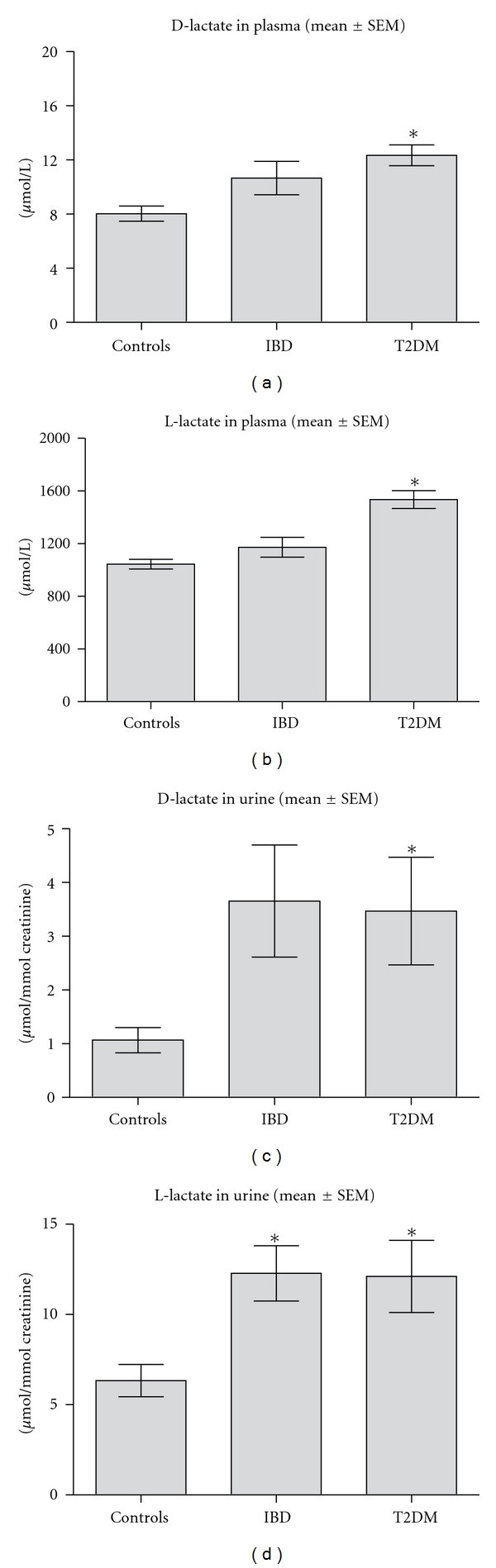
Urinary and plasma D- and L-Lactate concentrations of controls, patients with inflammatory bowel disease (IBD), and patients with type 2 diabetic patients (T2DM). Data are presented as Mean ± SEM: (*P < 0.05).

We next investigated D- and L-Lactate plasma and urine concentration of nondiabetic controls, patients with IBD, and T2DM patients (Figure 3). In healthy controls, D- and L-Lactate concentrations were 8.0 ± 0.6 and 1044.8 ± 36.7 *μ*mol/L in plasma, respectively, and 1.1 ± 0.2 and 6.3 ± 0.9 **μ**mol/mmol creatinine in urine, respectively (mean ± SEM). In IBD patients, levels of D- and L-Lactate were higher, when compared to the nondiabetic control group in both plasma (10.7 ± 1.2 and 1172.4 ± 74.6 *μ*mol/L, resp.) and urine (3.1 ± 0.8 and 11.8 ± 1.4 *μ*mol/mmol creatinine, resp.), which was significant for urinary L-lactate.

 In T2DM patients, the concentrations of D- and L-Lactate were significantly higher in plasma (12.3 ± 0.8 and 1534.7 ± 67.5 *μ*mol/L, resp.) and urine (3.4 ± 1.0 and 12.1 ± 2.0 *μ*mol/mmol creatinine, resp.). Both plasma and urinary D-lactate levels, as determined in T2DM and controls, correlated with HbA1C (*r* = 0.392, *P* < 0.001 and = 0.421, *P* < 0.001, resp.) and fasting plasma glucose (*r* = 0.360, *P* < 0.001 and *r* = 0.416, *P* < 0.001, resp.).

## 4. Discussion

We describe here a rapid, sensitive, and highly specific method for the simultaneous determination of D- and L-Lactate in plasma and urine by UPLC MS/MS. The derivatisation of D- and L-Lactate with DATAN makes it possible to separate both enantiomers on a reversed-phase-based analytical column. This results in a robust chromatographic system without the need of a column-switching or solid phase extraction (SPE) presample cleanup. We found that urinary and plasma levels of D- and L-Lactate were significantly increased in T2DM as compared with nondiabetic controls. In addition, we observed higher L- and D-Lactate levels in patients with IBD, but only significant for urinary L-lactate.

Many other techniques have been used to quantify D- and L-Lactate, with several disadvantages, such as long run times [3, 11, 26, 27], large sample volume [19, 21, 22, 24, 27], a column-switching preseparation technique [3, 12, 13], or low sensitivity [11, 24, 27]. Moreover, a disadvantage of the enzymatic method is that it is not possible to measure D- and L-Lactate in a single run. 

Chiral stationary phase liquid chromatography has been applied for the enantiomeric separation of D- and L-Lactate [11, 14, 25]. SPE or prereversed phase liquid chromatographic separation is obligatory for good chiral chromatographic performance [3, 12, 13, 31]. Furthermore, the shorter lifetime, higher cost, and difficult selection of a suitable chiral column [32, 33] have made an alternative method for enantiomeric separation desirable. More recently, Cevasco et al. [26] used a reversed phase liquid chromatography method for separation of D- and L-lactic acid. However, a run-to-run time of 35 minutes and an obligatory SPE sample preparation step make this method less feasible for large cohort studies. Moreover, the used derivatisation reagent was not commercially available and had to be synthesised before use.

Anhydrides of tartaric acid were used successfully as chiral derivatisation reagents of hydroxy acids and other enantiomeric compounds [34–36]. In this paper, we describe the derivatisation of the enantiomeric D- and L-Lactate with DATAN. This derivatisation step results in a highly sensitive and specific D- and L-Lactate derivative which is baseline separated on a UPLC reversed phase column and detected with tandem MS. The advantage of this technique, as compared to the described methods in the literature, is that there is no need for sample cleanup or preseparation of the sample matrix, and only 25 *μ*L of sample is necessary. Also the highly efficient and specific fragments of these DATAN derivatives generated in the collision cell is an improvement against the nonderivatized analysis of D- and L-Lactate with LC/MS [25]. With a run-to-run time of 6 minutes we established a fast and reliable method suitable for measuring D- and L-Lactate in large cohort studies.

The D- and L-Lactate concentrations we measured in plasma and urine from healthy controls are in reasonable agreement with data obtained by other techniques [3, 18, 19, 25]. Indeed, we found an acceptable correlation of the new UPLC tandem MS method with the enzymatic assay in urine samples. However, the enzymatic assay is not adequately sensitive for lower levels of D-lactate, as reflected in the Bland-Altman plot. The enzymatic method measures higher levels of D-lactate than the UPLC tandem MS in the lower range.

D-lactate was not significantly increased in patients with IBD compared with nondiabetic controls in both plasma and urine. Another study, however, found a significant increase of D-lactate in hospitalised patients with active IBD [6]. This difference may be explained by the fact that the patients we have included were in remission. In addition, due to the relatively small sample size, the power to detect statistically differences was low.

We found a statistically significant increase of urine and plasma levels of D- and L-Lactate in T2DM as compared with nondiabetic controls. The fact that both D-lactate and L-lactate are increased in patients with T2DM suggests that the hyperglycaemic state is an important source of D-lactate elevations in diabetes. L-lactate is mainly formed during glycolysis by conversion of pyruvate to L-lactate by lactate dehydrogenase. D-lactate is an endproduct of the metabolism of methylglyoxal, formed during hyperglycaemia, by the glyoxalase pathway [37]. In line with this, we demonstrate that D-lactate correlates significantly with HbA1C, a marker for prolonged hyperglycaemia. However, based on our small study, we cannot definitely conclude whether D-lactate is merely a reflection of methylglyoxal, gut-flora, or both, as several possible residual confounding factors such as BMI and gut permeability may explain the differences we observed between our patient groups.

Methylglyoxal is produced in small amount from carbohydrates, fat, and protein metabolism. It is has been demonstrated that methylglyoxal is the most important precursor in the formation of advanced glycation endproducts. Methylglyoxal and methylglyoxal-derived advanced glycation endproducts are believed to be implicated in the development of diabetic vascular complications. Because D-lactate is elevated in diabetes and may be used as an indicator of methylglyoxal, the measurement of D-lactate needs to be evaluated in cohort studies with D-lactate as a possible predictor of diabetic complications. In addition, mechanistic studies are needed to elucidate the relative contribution of several metabolic pathways to the total urinary and plasma D-lactate pool, in both healthy and diabetic individuals.

In conclusion, this specific measurement of D- and L-Lactate shows promise in the investigation of diabetes and metabolic diseases.

## Figures and Tables

**Table 1:** MRM settings.

Component	Parent ion (m/z)	Daughter ion (m/z)	Collision energy (eV)	Dwell (secs)
[^13^C_3_]-L-lactate	307.95	91.95	8.0	0.1
D-lactate	304.95	88.95	8.0	0.1
L-lactate	304.95	88.95	8.0	0.1

**Table 2:** Linearity tested in different matrices.

Matrix	Slope^(a)^	*Y*-intercept	*r* ^2^	Concentration range (*μ*mol/L)
D-lactate				
Water	1.2927	−4.5	0.9971	0–105
Plasma A	1.4307	21.2	0.9987	0–105
Plasma B	1.3820	51.3	0.9991	0–105
Plasma C	1.5037	31.9	0.9990	0–105

Mean	1.4023			
CV(%)	6.3			
L-lactate				
Water	0.7768	−4.9	0.9996	0–3008
Plasma A	0.7566	1032	0.9998	0–6016
Plasma B	0.7164	996	0.9997	0–6016
Plasma C	0.7534	1346	0.9997	0–3008

Mean	0.7502			
CV(%)	3.2			
D-lactate				
Water	1.1265	−15.4	0.9999	0–702
Urine A	1.1092	105.24	0.9984	0–351
Urine B	1.1114	99.7	0.9999	0–702
Urine C	1.0287	69.8	0.9992	0–351

Mean	1.094			
CV(%)	4.04			
L-lactate				
Water	0.7967	0.2	0.9998	0–1002
Urine A	0.8039	60.4	0.9995	0–501
Urine B	0.7918	48.6	0.9995	0–1002
Urine C	0.7749	135.0	0.9993	0–501

Mean	0.7967			
CV(%)	1.56			

^(a)^Slope: {concentration (*μ*mol/L)} versus {response = (peak area component/peak area internal standard). ∗Internal standard concentration (*μ*mol/L)}.

**Table 3:** Recovery data for plasma and urine.

Plasma	Mean (SD) *μ*mol/L	CV, %	Recovery, %
D-lactate added^(a)^ *μ*mol/L (*n* = 5)			
0	8.1 (0.2)	2.9	—
52.7	67.3 (1.2)	1.8	112.4
105.4	127.7 (3.7)	2.9	113.5

L-lactate added^(a)^ *μ*mol/L (*n* = 5)			
0	1365 (44.6)	3.3	—
1504	2931 (26.2)	0.9	104.2
3008	4443.2 (125.1)	2.8	102.3

Urine^(b)^	Mean (SD) *μ*mol/mmol creatinine	CV, %	Recovery, %

D-lactate added^(a)^ *μ*mol/L (*n* = 5)			
0	0.91 (0.03)	3.4	—
98.8	8.03 (0.10)	1.3	110.9
197.6	14.34 (0.29)	2.0	104.7

L-lactate added^(a)^ *μ*mol/L (*n* = 5)			
0	4.84 (0.13)	2.6	—
94	10.99 (0.38)	3.5	100.6
188	16.56 (0.14)	0.8	96.0

^(a)^Addition of 25 *μ*L standard solution to 25 *μ*L plasma or urine.

^(b)^Creatinine concentration: 15.4 mmol/L.

**Table 4:** Precision data for D- and L-Lactate in plasma and urine.

Matrix	D-lactate mean (SD), *μ*mol/L	CV,%	L-lactate mean (SD), *μ*mol/L	CV,%
Intra-assay, *n* = 10				
Plasma A	13.0 (0.7)	5.1	1265.3 (36.2)	2.9
Plasma B	85.7 (2.5)	2.9	6605.8 (190.4)	2.9

Interassay, *n* = 10				
Plasma A	12.4 (0.6)	5.2	1338.7 (48.8)	3.6
Plasma B	85.4 (3.8)	4.4	6452.6 (245.8)	3.8

	D-lactate mean (SD), *μ*mol/mmol creatinine		L-lactate mean (SD), *μ*mol/mmol creatinine	CV, %

Intra-assay, *n* = 10				
Urine A^(1)^	0.857 (0.03)	3.8	4.40 (0.27)	6.0
Urine B^(2)^	16.26 (0.48)	2.9	10.58 (0.60)	5.7

Interassay, *n* = 10				
Urine A^(1)^	0.718 (0.04)	5.6	3.75 (0.33)	8.8
Urine B^(2)^	14.90 (1.05)	7.0	8.68 (0.81)	9.3

^(1)^Urine A, creatinine 15.4 mmol/L.

^(2)^Urine B, creatinine 5.9 mmol/L.

## Abbreviations

CTMM: The Center for Translational Molecular Medicine
IBD: Inflammatory bowel disease
T2DM: Type 2 diabetes
GC/MS: Gas chromatography mass spectrometry
LC/MS: Liquid chromatography mass spectrometry
UPLC: Ultraperformance liquid chromatography
MS/MS: Tandem mass spectrometry
DATAN: (+)-O,O'-diacetyl-L-tartaric anhydride
MRM: Multiple-reaction-monitoring
SEM: Standard error of the mean
ANOVA: Analysis of variance
SPE: Solid phase extraction.
